# The mitochondrial alternative oxidase pathway protects the photosynthetic apparatus against photodamage in *Rumex *K-1 leaves

**DOI:** 10.1186/1471-2229-12-40

**Published:** 2012-03-20

**Authors:** Li-Tao Zhang, Zi-Shan Zhang, Hui-Yuan Gao, Xiang-Long Meng, Cheng Yang, Jian-Guo Liu, Qing-Wei Meng

**Affiliations:** 1 State Key Laboratory of Crop Biology, Shandong Agricultural University, Tai'an 271018, Shandong, China; 2 Institute of Oceanology, Chinese Academy of Sciences, Qingdao 266071, Shandong, China

## Abstract

**Background:**

It is known that excess reducing equivalents in the form of NADPH in chloroplasts can be transported via shuttle machineries, such as the malate-oxaloacetate (OAA) shuttle, into the mitochondria, where they are efficiently oxidised by the mitochondrial alternative oxidase (AOX) respiratory pathway. Therefore, it has been speculated that the AOX pathway may protect plants from photoinhibition, but the mechanism by which this protection occurs remains to be elucidated.

**Results:**

The observation that the malate-OAA shuttle activity and the AOX pathway capacity increased markedly after intense light treatment in *Rumex *K-1 leaves indicates that excess NADPH was transported from the chloroplasts and oxidised by the AOX pathway. The inhibition of the AOX pathway by salicylhydroxamic acid (SHAM) caused the over-reduction of the photosystem I (PSI) acceptor side, as indicated by the increases in the extent of reduction of P700^+^. Furthermore, the photosynthetic linear electron flow was restricted, which was indicated by the decreases in the PSII electron transport rate (ETR) and the photosynthetic O_2 _evolution rate. The restriction of the photosynthetic linear electron flow, which generates the thylakoid ΔpH, inevitably decreased the de-epoxidation of the xanthophyll cycle (ΔPRI). Therefore, the induction of non-photochemical quenching (NPQ) was suppressed when the AOX pathway was inhibited. The effect of the inhibition of the AOX pathway on NPQ induction was less at 20 mM NaHCO_3 _than at 1 mM NaHCO_3_. The suppression of NPQ induction by the inhibition of the AOX pathway was also observed during the induction phase of photosynthesis. In addition, the inhibition of the AOX pathway increased the accumulation of hydrogen peroxide (H_2_O_2_), suggesting that the AOX pathway functions as an antioxidant mechanism.

**Conclusions:**

The inhibition of the AOX pathway resulted in the rapid accumulation of NADPH in the chloroplasts, which caused the over-reduction of the PSI acceptor side. Furthermore, the restriction of the photosynthetic linear electron flow due to the inhibition of the AOX pathway limited the generation of the thylakoid ΔpH and suppressed the induction of NPQ. Therefore, the mitochondrial AOX pathway protected the photosynthetic apparatus against photodamage by alleviating the over-reduction of the PSI acceptor side and accelerating the induction of NPQ in *Rumex *K-1 leaves.

## Background

Excess light energy will result in the accumulation of reducing equivalents in the form of NADPH generated by photochemical reactions. The accumulation of the reducing equivalents in the chloroplasts causes the over-reduction of the photosynthetic electron transport chain and accelerates the generation of reactive oxygen species (ROS), leading to the disruption of the photosynthetic apparatus (photoinhibition) [1-3]. However, because most plants cannot escape from exposure to excess light, they have evolved various defence mechanisms to dissipate excess light energy, such as non-photochemical quenching (NPQ) [3-7], cyclic electron flow around PSI/II (CEF-PSI/II) [8-12] and the water-water cycle (WWC) [13-15]. Though such intra-chloroplastic defence systems have been studied extensively, little is known about the extra-chloroplastic defence systems [16].

**Figure 1:**
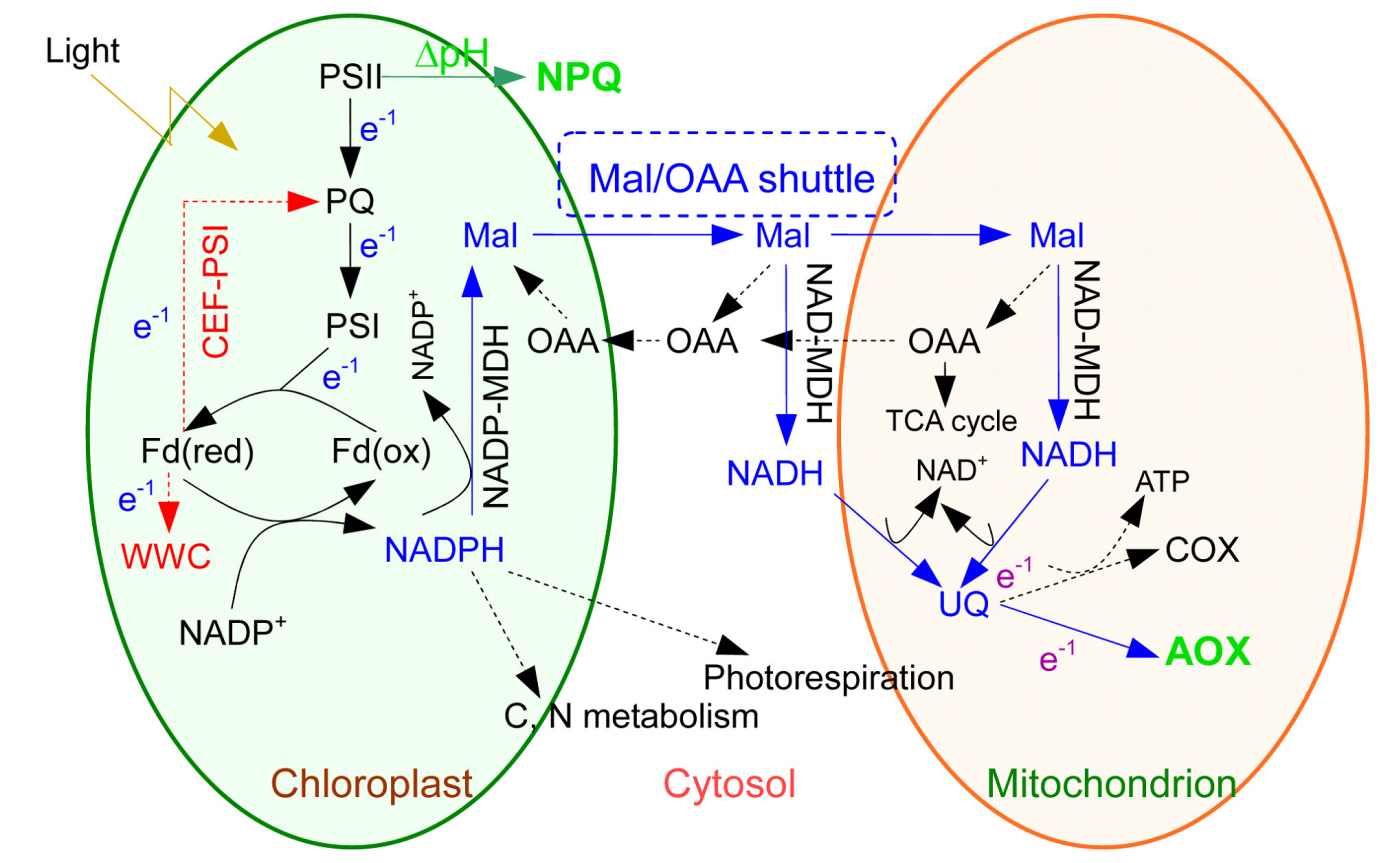
**The photosynthetic electron sinks in higher plants**. AOX, alternative oxidase; CEF-PSI, cyclic electron flow around PSI; COX, cytochrome oxidase; Fd, ferredoxin; Mal, malate; NAD(P)-MDH, NAD(P)-malate dehydrogenase; NPQ, non-photochemical quenching; OAA, oxaloacetate; PQ, plastoquinone; PSII/I, photosystem II/I; UQ, ubiquinone; WWC, water-water cycle.

It has been shown that excess reducing equivalents in the form of NADPH generated by photosynthesis can be transported to the cytosol, peroxisomes and mitochondria via shuttle machineries, such as the malate-oxaloacetate (OAA) shuttle [17-20], where they are oxidised in metabolic pathways under photoinhibitory conditions. In the mitochondria, the excess reducing equivalents can be oxidised by the respiratory electron transport chain. The respiratory electron transport in the mitochondria of higher plants uses two different pathways, the cyanide-sensitive cytochrome oxidase (COX) pathway and the cyanide-resistant alternative oxidase (AOX) pathway [18,21]. The COX pathway accomplishes most of the ATP production in the plant mitochondria, whereas proton translocation and ATP synthesis are uncoupled in the AOX pathway [22-24]. Therefore, the AOX pathway is a non-phosphorylating pathway and can efficiently oxidise the reducing equivalents generated in the chloroplasts without being restricted by the proton gradient across the mitochondrial inner membrane or the cellular ATP/ADP ratio. In fact, several studies have demonstrated that the AOX pathway functions as a sink for the excess reducing equivalents generated by photosynthesis (Figure 1, blue arrows) [16,25]. Therefore, it has been speculated that the AOX pathway may play a particular role in protecting plants from photoinhibition [16,18,25-28]; however, the exact mechanism by which the mitochondrial AOX pathway alleviates photoinhibition remains to be elucidated.

As described above, NPQ acts as a mechanism to protect plants from the damage caused by excess light energy, which has been studied for many years [1-3]. However, most of the studies on NPQ have focused on the regulations and molecular mechanisms, emphasising the pivotal roles of the trans-thylakoid membrane proton gradient (ΔpH) and the xanthophyll cycle [4-7]. Less attention has been paid to the question of the type of electron flow that is responsible for the formation of the thylakoid ΔpH, which drives the conversion of violaxanthin to zeaxanthin via the intermediate antheraxanthin [13].

The induction of NPQ requires a thylakoid ΔpH generated by photosynthetic electron transport to activate the de-epoxidation of violaxanthin to zeaxanthin [3,7]. However, under intense light conditions, the photosynthetic electron transport system becomes filled up with electrons due to the accumulation of excess reducing equivalents and the insufficient regeneration of both NADP^+ ^and ADP [2,9,14]. Under such conditions, alternative electron sinks play important roles in the maintenance of the photosynthetic electron transport chain, resulting in a buildup of the thylakoid ΔpH.

In previous studies, two alternative electron sinks are proposed for the induction of NPQ through the formation of the thylakoid ΔpH (Figure 1, red arrows). The first such sink is the cyclic electron flow around PSI (CEF-PSI) [8,9,11,12,29]. Miyake et al. [9] have already reported that CEF-PSI activity is required as an alternative electron flow to produce the thylakoid ΔpH and synthesise more ATP for CO_2 _assimilation. Meanwhile, these researchers demonstrate that CEF-PSI always has a higher activity than that required for CO_2 _assimilation, especially when the plants are under high light intensity and at a low concentration of CO_2_. They prove that CEF-PSI contributes to the formation of the thylakoid ΔpH, driving the de-epoxidation of violaxanthin and leading to the strong induction of NPQ. The second alternative electron pathway is the water-water cycle (WWC) [11,13,15,30]. When the electron flux in PSII exceeds the total electron flux required for CO_2 _assimilation and photorespiration, reducing equivalents are rapidly generated by the photochemical reaction in the form of NADPH. Molecular oxygen may then substitute as an electron acceptor in the WWC to allow the linear electron flow to continue, forming a ΔpH across the thylakoid membranes to drive the de-epoxidation of violaxanthin. Therefore, the WWC plays a central role in the induction of NPQ.

The CEF-PSI and the WWC are two major alternative electron flows to generate the thylakoid ΔpH and lead to the induction of NPQ in the chloroplasts. As described above, it has also been suggested that the mitochondrial AOX pathway functions as an alternative sink for the excess electrons generated by photosynthesis (Figure 1, blue arrows), similarly to the CEF-PSI and the WWC (Figure 1, red arrows). It remains unclear whether the mitochondrial AOX pathway also contributes to the thylakoid ΔpH to induce the induction of NPQ as an extra-chloroplastic sink for the electrons. In the present work, we examined the effects of the inhibition of the AOX pathway by various concentrations of SHAM on the chlorophyll *a *fluorescence transient, the photosynthetic fluorescence parameters, the de-epoxidation of the xanthophyll cycle (ΔPRI) and the induction of NPQ in *Rumex *K-1 leaves. The physiological function of the AOX pathway in the induction of NPQ during the steady state and the induction phase of photosynthesis was confirmed, and the photoprotection mechanisms of the AOX pathway are discussed. The *Rumex *K-1 used in this study, a hybrid of *Rumex patientia *× *R. tianschaious*, is a salt-tolerant fodder crop with a high content of leaf protein that is used in northwestern China for the reclamation of saline soil.

## Results

### The effects of SHAM treatments on Φ_PSII_, qP and NPQ in the isolated intact *Rumex *K-1 chloroplasts

**Figure 2:**
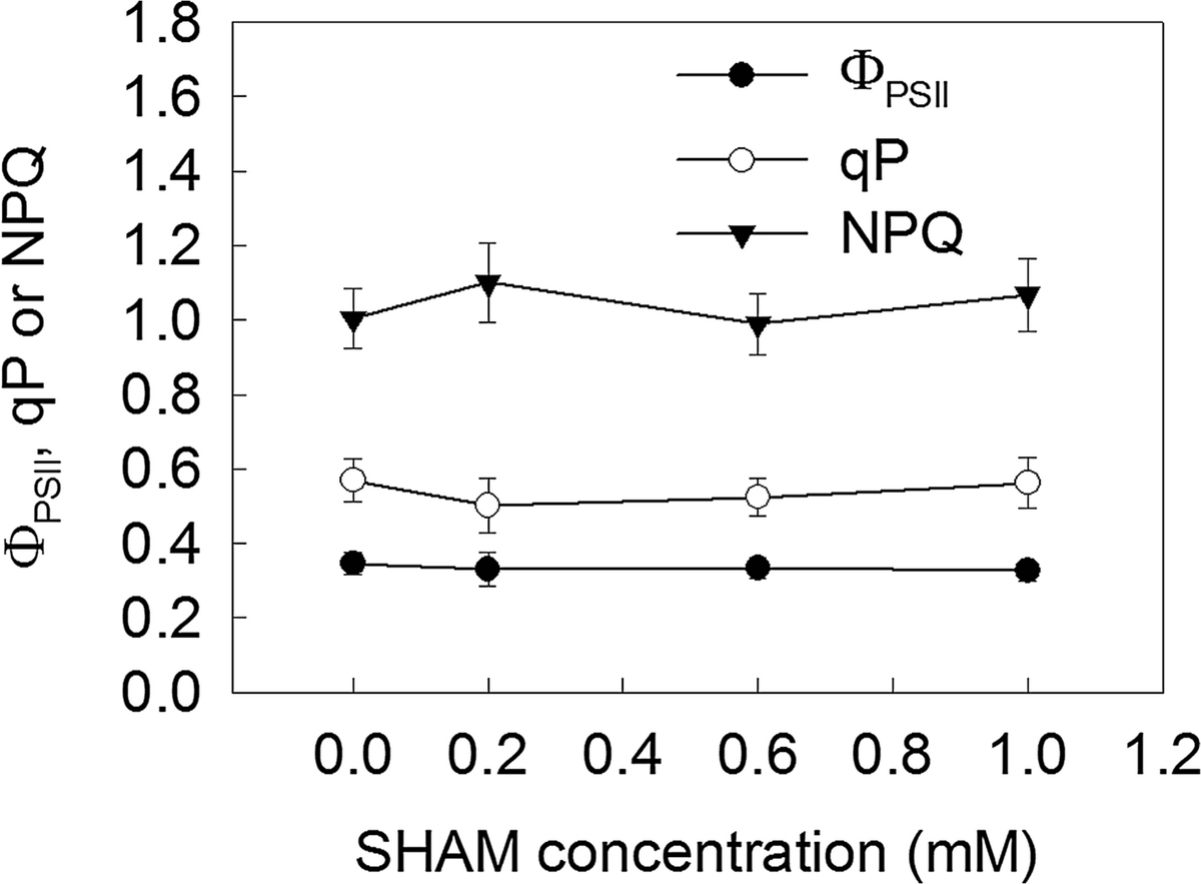
**Φ_PSII_, qP and NPQ in the isolated *Rumex *K-1 chloroplasts**. Chloroplasts were incubated in darkness for 10 minutes in the presence of 0 (control), 0.2, 0.6 or 1 mM SHAM and afterward irradiated with 800 μmol m^-2 ^s^-1^. Means ± SE of ten replicates are presented.

Salicylhydroxamic acid (SHAM) has been widely used to inhibit the AOX pathway [26,31-33]. Given that several components of the photosynthetic electron transport chain in the chloroplasts are similar to those in the respiratory chain of the mitochondria, we examined whether the concentrations of SHAM used in this study had direct effects on the photosynthetic electron transport chain. The results demonstrated that the treatments with 0 (control), 0.2, 0.6 or 1 mM SHAM had no direct effects on the actual PSII photochemical efficiencies (Φ_PSII_), the photochemical quenching coefficients (qP) or the non-photochemical quenching (NPQ) in intact chloroplasts isolated from *Rumex *K-1 leaves (Figure 2), suggesting that the concentrations of SHAM used in this study had no direct effects on photosynthetic behaviours.

### The effects of SHAM treatments on the AOX pathway capacities and the NADP-malate dehydrogenase (NADP-MDH) initial activities in *Rumex *K-1 leaves

**Figure 3:**
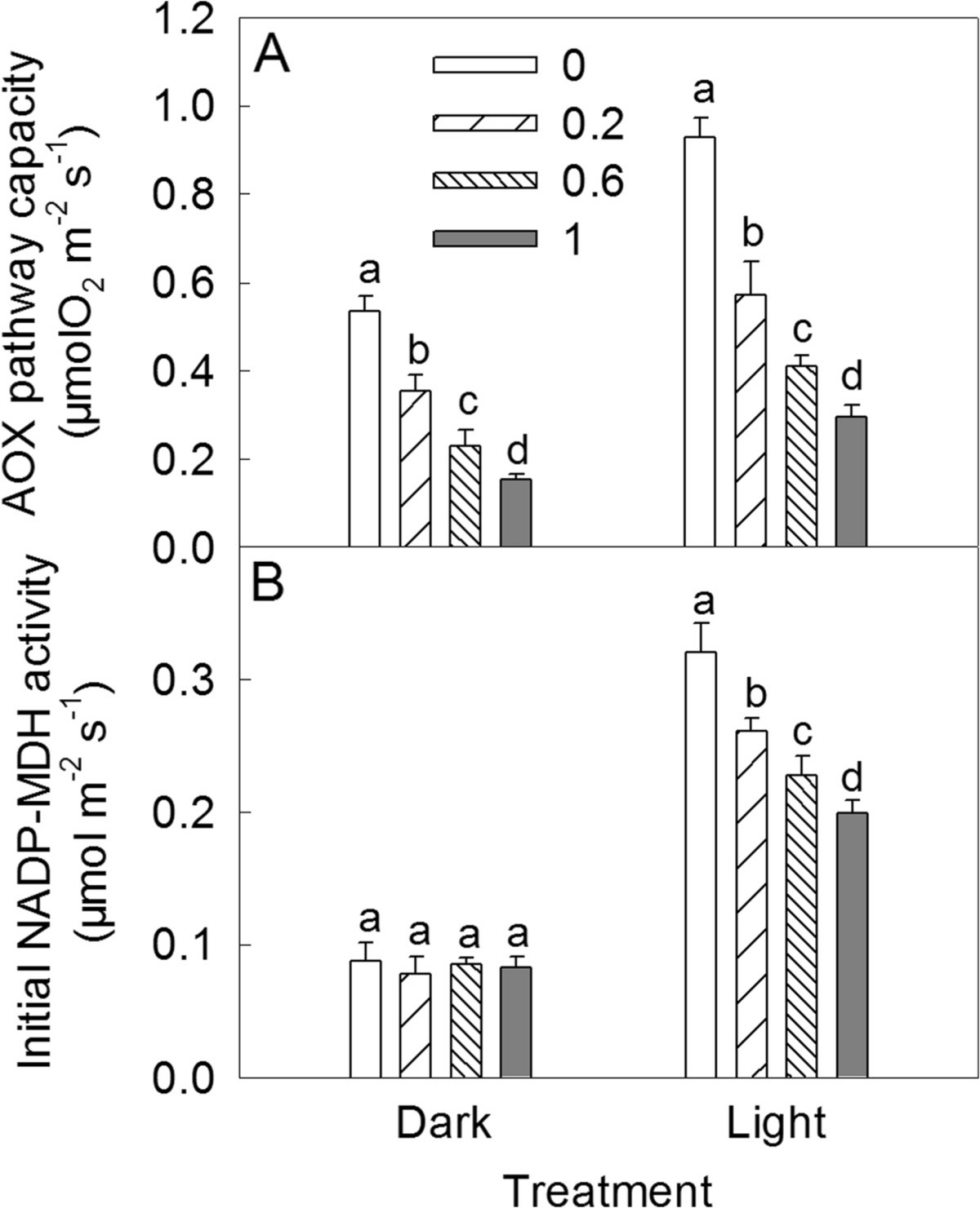
**The AOX pathway capacities (A) and the NADP-MDH initial activities (B) in *Rumex *K-1 leaves treated with 0 (control), 0.2, 0.6 or 1 mM SHAM in the dark or in intense light**. Leaf dics (0.5 cm^2^) were infiltrated with 0 (control), 0.2, 0.6 or 1 mM SHAM in the dark for 2 h and then exposed to intense light measuring 800 μmol m^-2 ^s^-1 ^for 30 minutes. Means ± SE of five replicates are presented. Different letters indicate significant difference between the treatments with various concentrations of SHAM at *P *< 0.05, the same as this in other figures.

To determine a working concentration of the inhibitor for this study, we examined the effects of the inhibitor on the capacities of the AOX pathways in *Rumex *K-1 leaves in the dark or in intense light. Figure 3 A shows that the AOX pathway capacity increased significantly after intense light treatment of the control leaves. Treatments with 0.2, 0.6 and 1 mM SHAM inhibited approximately 34%, 57%, and 71% and 38%, 56%, 68% of the AOX pathway capacities in the dark and in the intense light, respectively. The initial activity of NADP-malate dehydrogenase (NADP-MDH) increased by approximately 265% after intense light treatment of the control leaves. The SHAM treatments did not alter the initial activities of NADP-MDH in the dark. However, under intense light, the initial activities of NADP-MDH decreased by approximately 19%, 29% and 38% in *Rumex *K-1 leaves treated with 0.2, 0.6 and 1 mM SHAM, respectively (Figure 3 B).

### The effects of SHAM treatments on the chlorophyll *a *fluorescence transients and the P700 redox state in *Rumex *K-1 leaves

The chlorophyll *a *transient has become one of the most popular tools in photosynthetic research [34-38]. The shape of the OJIP transient is very sensitive to environmental stresses. Strasser and Strasser [39] developed a procedure to quantify the OJIP transient, known as the JIP-test. With this test, it is possible to calculate several phenomenological and biophysical expressions of the photosynthetic electron transport chain [39-41]. The JIP-test is thus a powerful tool for the in vivo investigation of photosynthetic behaviours, including the energy fluxes of absorption, trapping and electron transport [40,41]. In the present study, we used the chlorophyll *a *fluorescence transient (OJIP) to detect changes in photosynthetic behaviours when the AOX pathway was inhibited.

**Figure 4:**
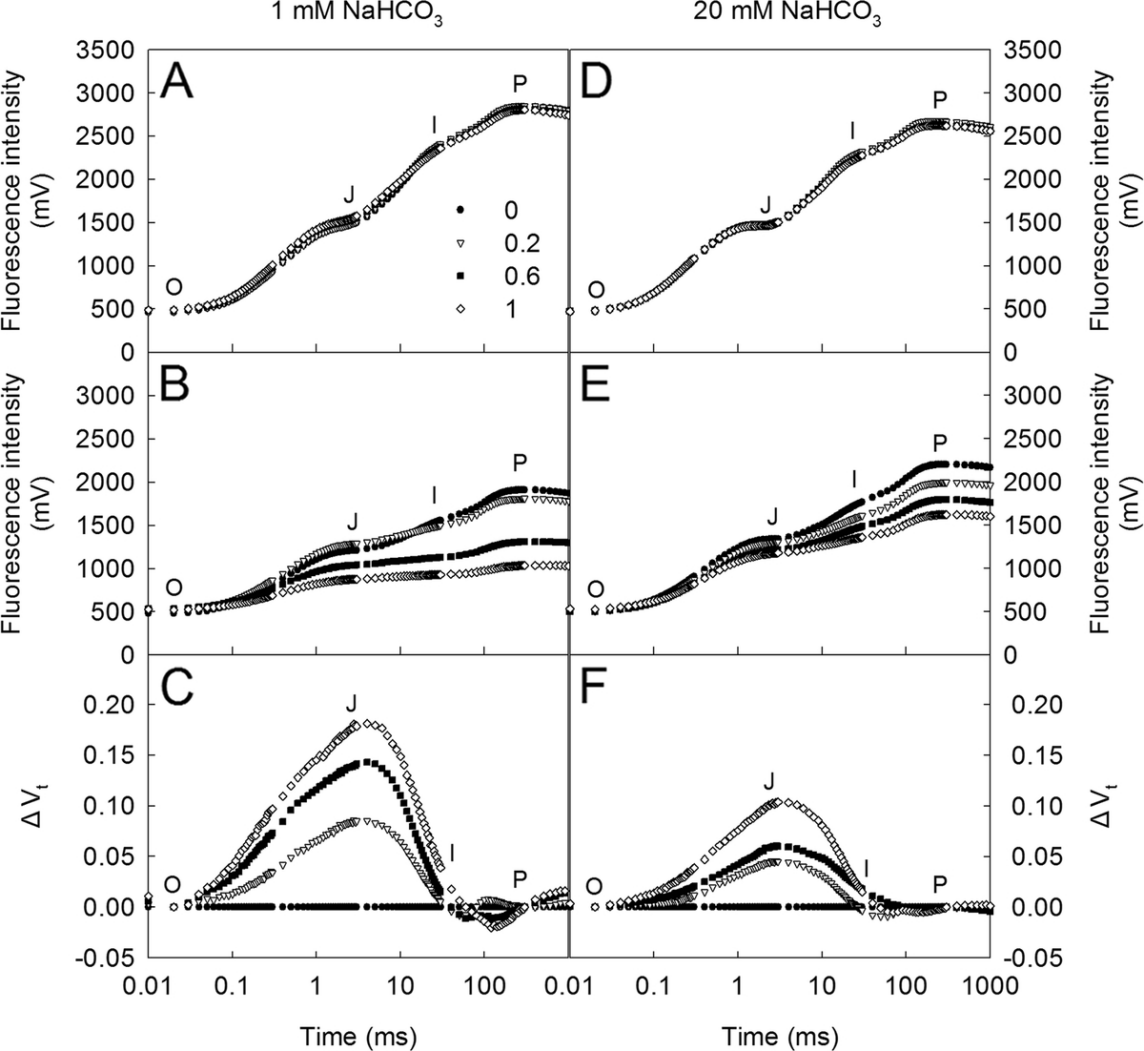
**The changes in the OJIP transients in *Rumex *K-1 leaves treated with 0 (control), 0.2, 0.6 or 1 mM SHAM in the dark (A, D) or in intense light (B, E) in the presence of 1 mM (A, B) or 20 mM (D, E) NaHCO_3_**. Treatments are as described in Figure 3. Each transient represents the average of twenty replicates.

Before intense light treatment, both the control and the SHAM-treated leaves showed a typical OJIP transient in the presence of both 1 mM (Figure 4 A) and 20 mM (Figure 4 D) NaHCO_3_. However, the intense light-treatment markedly lowered the fluorescence intensity and altered the shapes of the OJIP transients in both the control and the SHAM-treated leaves in the presence of 1 mM NaHCO_3 _(Figure 4 B). When the transients were presented in the form of ΔV_t _(the difference in the kinetics of fluorescence transients between the control leaves and the SHAM-treated leaves) according to the JIP-test, it was observed that the relative variable fluorescence at the J-step (which occurred at approximately 2 ms) became distinct in the SHAM-treated leaves compared with the control leaves under intense light. Additionally, the J-step increased significantly with the increase in the SHAM concentration (Figure 4 C). However, the changes in the OJIP transients under intense light due to the inhibition of the AOX pathway were decreased in the presence of 20 mM NaHCO_3 _(Figure 4 E, F), which suggests that the high concentration of NaHCO_3 _markedly lowered the susceptibility of *Rumex *K-1 leaves to SHAM treatments.

**Figure 5:**
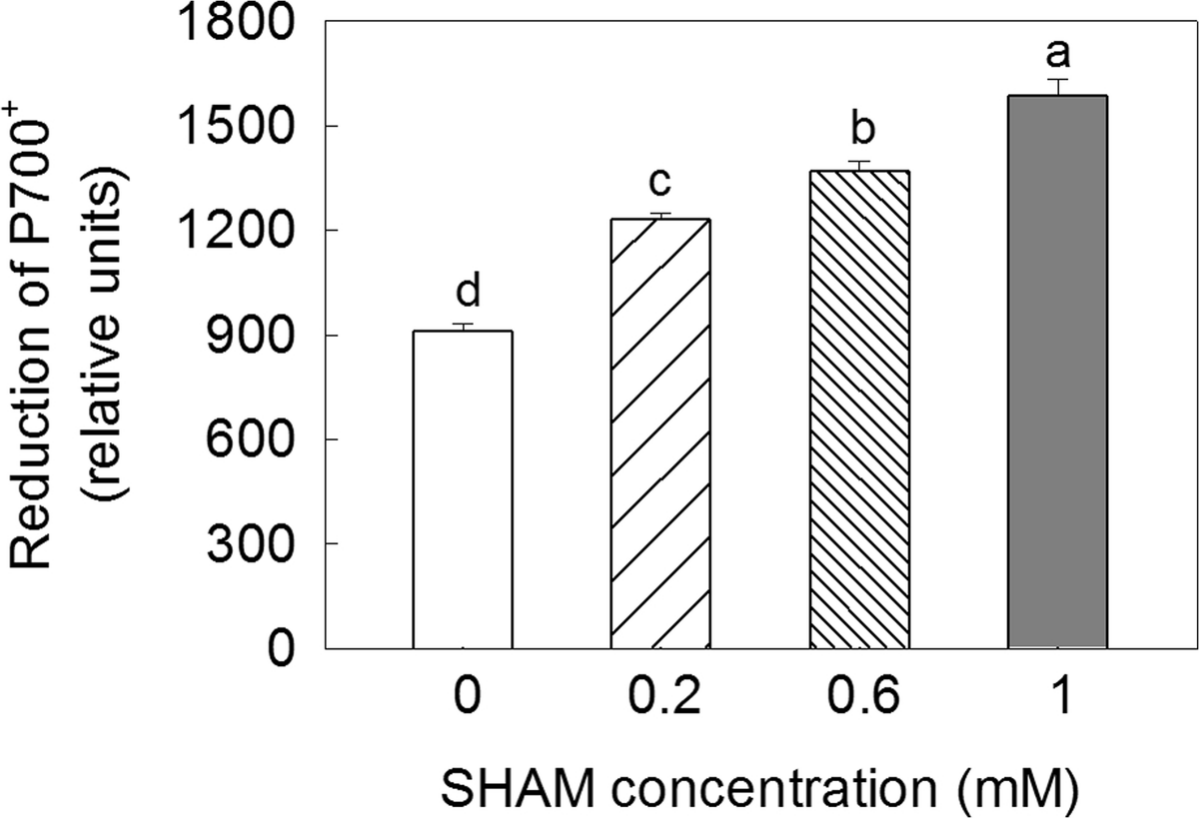
**The reduction of P700^+ ^in *Rumex *K-1 leaves treated with 0 (control), 0.2, 0.6 or 1 mM SHAM after exposure to intense light at 800 μmol m^-2 ^s^-1^**. Treatments are as described for Figure 3. Means ± SE of eight replicates are presented.

Furthermore, the extent of the reduction of P700^+ ^was more pronounced in the SHAM-treated leaves than in the control leaves, and the reduction extent of P700^+ ^increased with the increase in the SHAM concentration (Figure 5).

### The effects of SHAM treatments on the ETR, qP and photosynthetic O_2 _evolution rate in *Rumex *K-1 leaves

**Figure 6:**
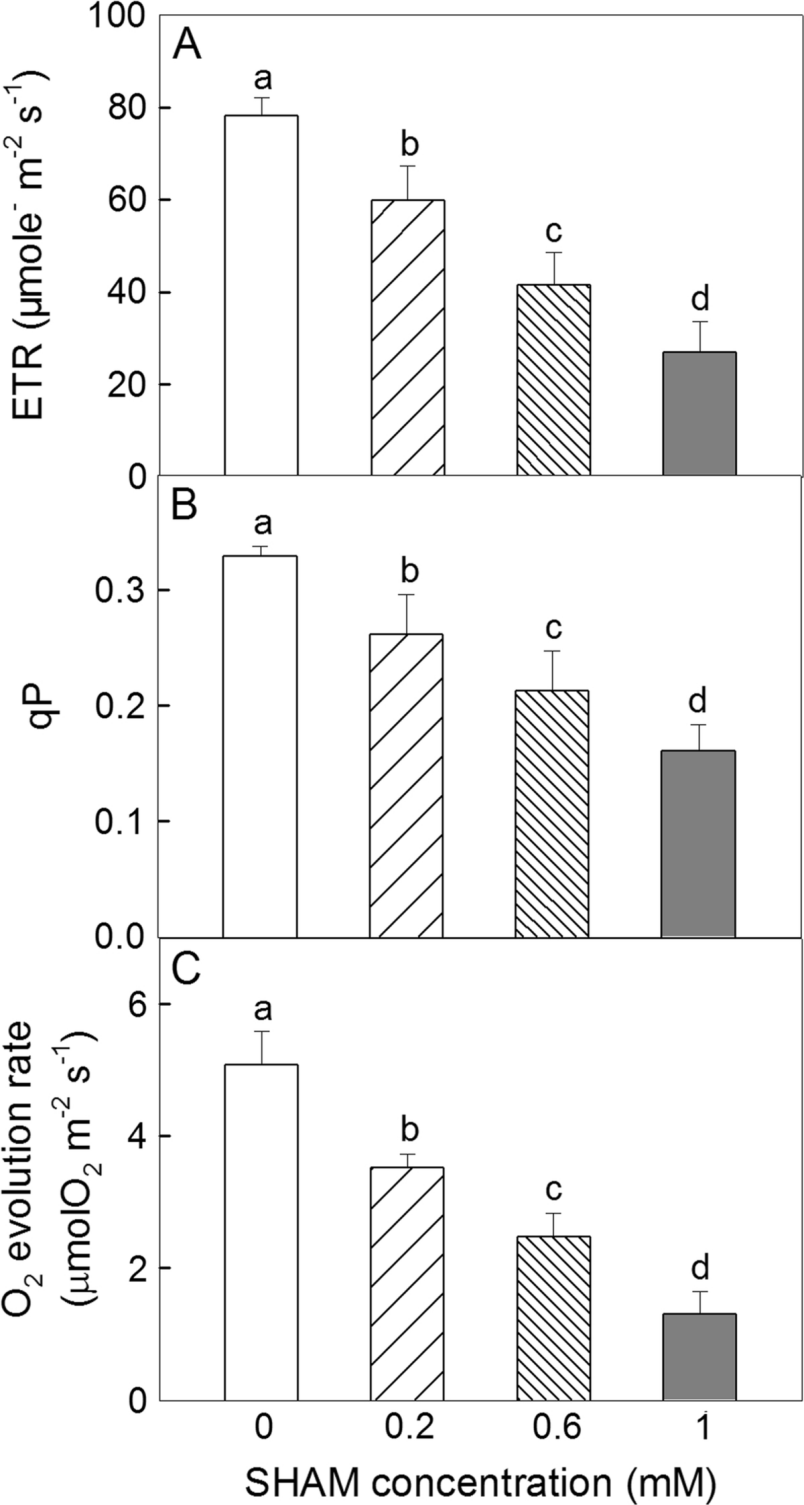
**The changes in the ETR (A), qP (B) and the photosynthetic O_2 _evolution rate (C) in *Rumex *K-1 leaves treated with 0 (control), 0.2, 0.6 or 1 mM SHAM**. Treatments are as described in Figure 3. Means ± SE of ten replicates are presented.

Changes in the PSII electron transport rate (ETR), the qP and the photosynthetic O_2 _evolution rate in *Rumex *K-1 leaves treated with various concentrations of SHAM are depicted in Figure 6. This figure shows that the ETR (Figure 6 A), the qP (Figure 6 B) and the photosynthetic O_2 _evolution rate (Figure 6 C) decreased significantly with the increase in the SHAM concentration.

### The effects of SHAM treatments on the ms-delayed light emission (ms-DLE), the de-epoxidation of the xanthophyll cycle (ΔPRI) and NPQ in *Rumex *K-1 leaves

**Figure 7:**
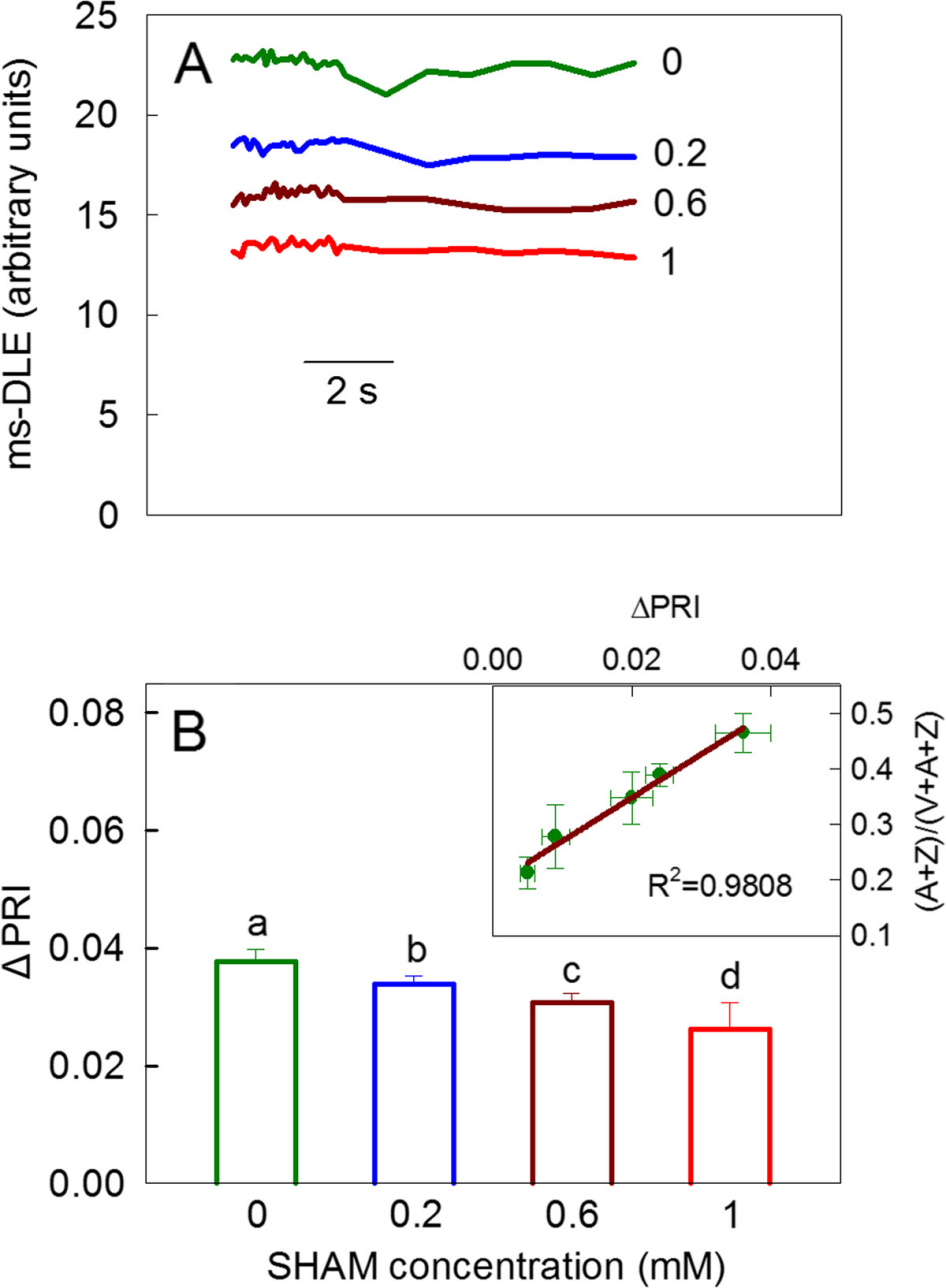
**The changes in the ms-DLE (A) and the ΔPRI (B) in *Rumex *K-1 leaves treated with 0 (control), 0.2, 0.6 or 1 mM SHAM**. The insert in Figure 7B was the correlation between ΔPRI and (A + Z)/(V + A + Z). Treatments are as described in Figure 3. Results are the average of five experiments at least.

The ms-delayed light emission (ms-DLE) of chlorophyll fluorescence has been used to monitor the thylakoid ΔpH [42,43]. The ms-DLE from the SHAM-treated leaf discs was lower than that from the control leaves after intense light treatment (Figure 7 A), suggesting that the inhibition of the AOX pathway restricted the formation of thylakoid ΔpH.

The actual de-epoxidation status of the xanthophyll cycle pigment pool ((A + Z)/(V + A + Z)) was positively correlated with the ΔPRI (the insert in Figure 7 B); therefore, the ΔPRI was used to estimate the de-epoxidation of the xanthophyll cycle in the experiment. Figure 7 B shows that the de-epoxidation of the xanthophyll cycle (ΔPRI) decreased significantly with the increase in the SHAM concentration in *Rumex *K-1 leaves. The ΔPRI in the leaves treated with 1 mM SHAM was approximately 69% of that in the control leaves.

**Figure 8:**
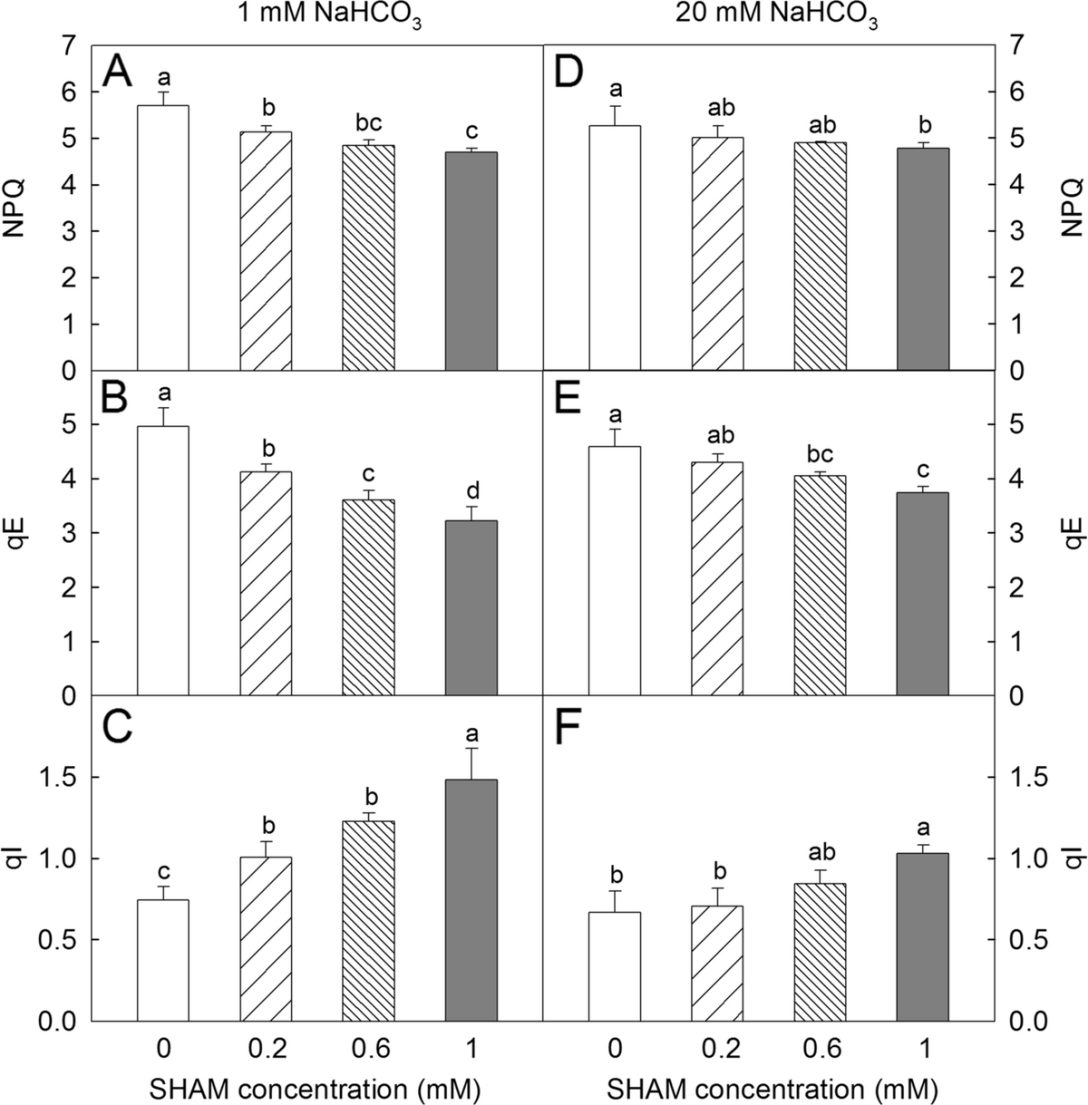
**The changes in NPQ (A, D), qE (B, E) and qI (C, F) in *Rumex *K-1 leaves treated with 0 (control), 0.2, 0.6 or 1 mM SHAM in the presence of 1 mM (A, B, C) or 20 mM (D, E, F) NaHCO_3_**. Treatments are as described in Figure 3. Means ± SE of ten replicates are presented.

Furthermore, with the increase in the SHAM concentration, NPQ and qE (fast component of NPQ) decreased, whereas qI (slow component of NPQ) increased in *Rumex *K-1 leaves in the presence of 1 mM and 20 mM NaHCO_3 _(Figure 8). Compared with the control leaves, NPQ and qE decreased by approximately 17% and 35%, respectively, and qI increased by approximately 99% in 1 mM SHAM-treated leaves in the presence of 1 mM NaHCO_3 _(Figure 8 A, B, C). This result showed that the contribution of qE to NPQ was more significant than that of qI. The changes in NPQ, qE and qI were less in the presence of 20 mM NaHCO_3_. The NPQ decreased by approximately 9%, the qE decreased by approximately 18%, and the qI increased by 54% in 1 mM SHAM-treated leaves compared with those in the control leaves in the presence of 20 mM NaHCO_3 _(Figure 8 D, E, F).

### The effects of SHAM treatments on the ETR, qP and NPQ during the induction phase of photosynthesis in *Rumex *K-1 leaves

**Figure 9:**
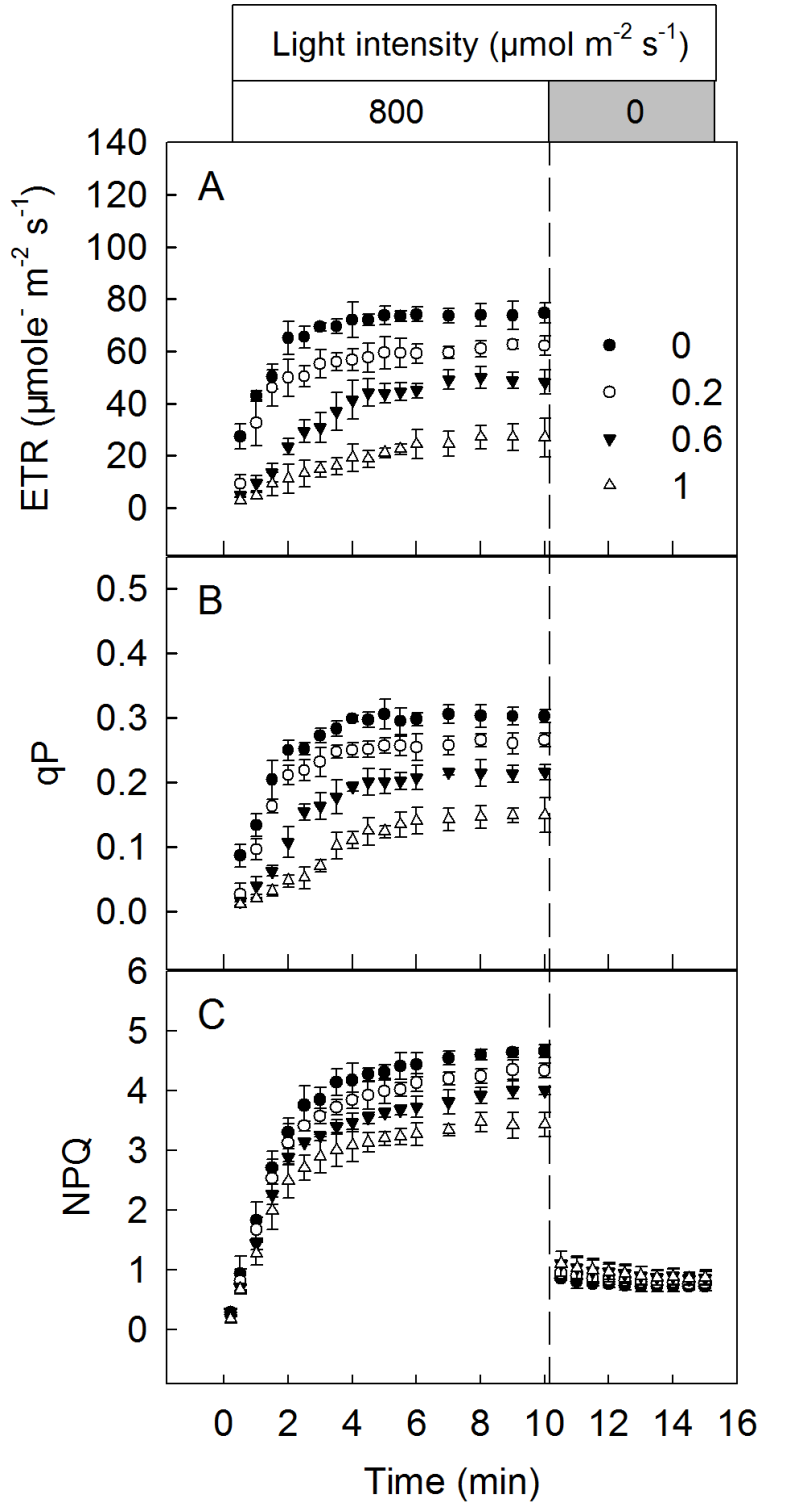
**The changes in the ETR (A), qP (B) and NPQ (C) during the induction phase of photosynthesis in *Rumex *K-1 leaves treated with 0 (control), 0.2, 0.6 or 1 mM SHAM**. Leaf discs were infiltrated with 0 (control), 0.2, 0.6 or 1 mM SHAM respectively in the dark for 2 h, then the induction and quenching kinetics of the ETR, qP and NPQ were measured. Means ± SE of five replicates are presented.

Figure 9 shows that the ETR and qP increased progressively during the first 5 minutes after exposure to intense light in both the control and the SHAM-treated leaves. However, the inhibition of the AOX pathway by SHAM restricted the increase of the ETR and qP after two minutes of illumination (Figure 9 A, B). The induction kinetics of NPQ exhibited a similar pattern to that of ETR and qP. The inhibition of the AOX pathway by SHAM also markedly restricted the development of NPQ after two minutes of illumination. After the leaves were transferred from the intense light to darkness, the NPQ was quenched within one minute, and the difference in the level of NPQ between various concentrations of SHAM-treated leaves was abolished (Figure 9 C).

### The effects of SHAM treatments on F_v_/F_m _in *Rumex *K-1 leaves

**Figure 10:**
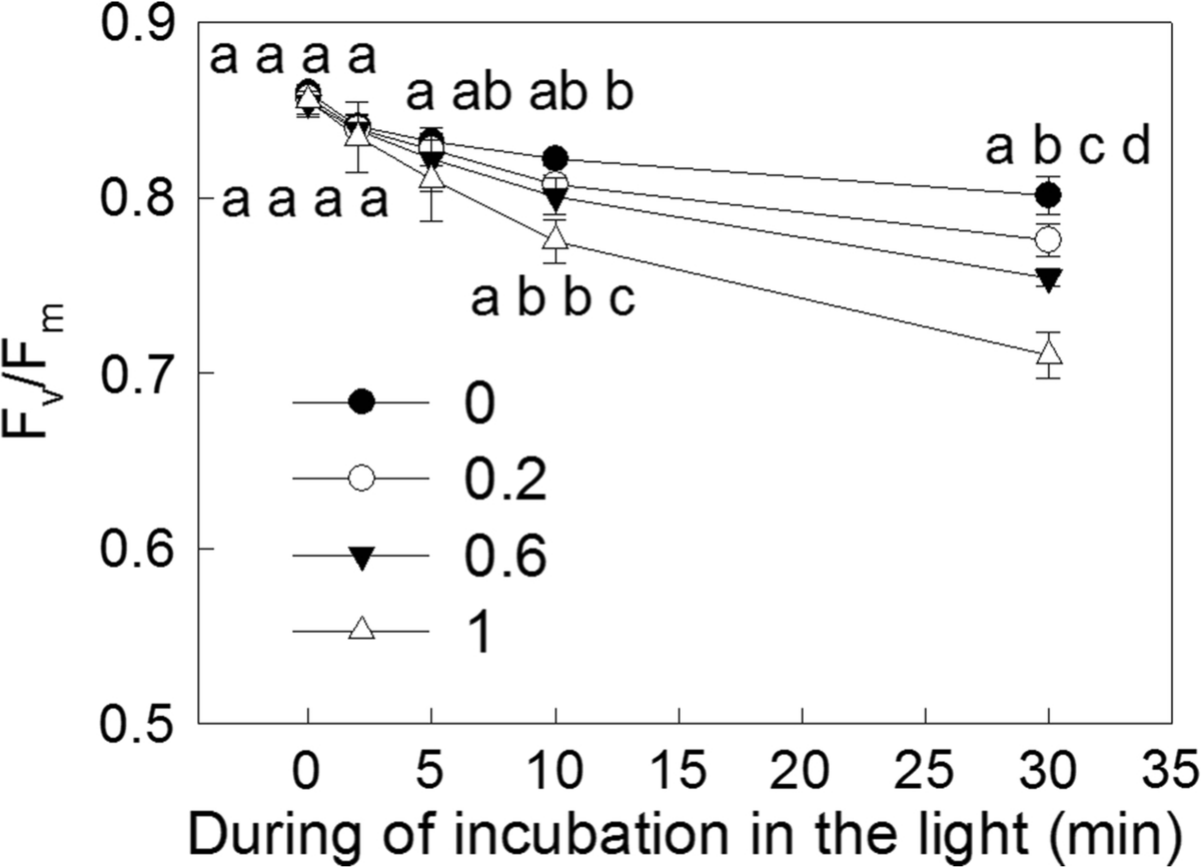
**F_v_/F_m _in *Rumex *K-1 leaves treated with 0 (control), 0.2, 0.6 or 1 mM SHAM after 0, 2, 5, 10 or 30 min of illumination**. The discs, adaxial side up, were exposed to intense light measuring 800 μmol m^-2 ^s^-1 ^for 0, 2, 5, 10, 30 minute respectively. Means ± SE of five replicates are presented.

To examine the effect of the inhibition of the AOX pathway on the photoinhibition of PSII, we measured the maximal quantum yield of PSII (F_v_/F_m_) in *Rumex *K-1 leaves after the leaves were transferred to intense light from darkness (Figure 10). The F_v_/F_m _in the control and the SHAM-treated leaves decreased after exposure to intense light, and the inhibition of the AOX pathway accelerated the decrease in F_v_/F_m _after 5 minutes of illumination.

### The effects of SHAM treatments on the hydrogen peroxide (H_2_O_2_) contents in *Rumex *K-1 leaves

**Figure 11:**
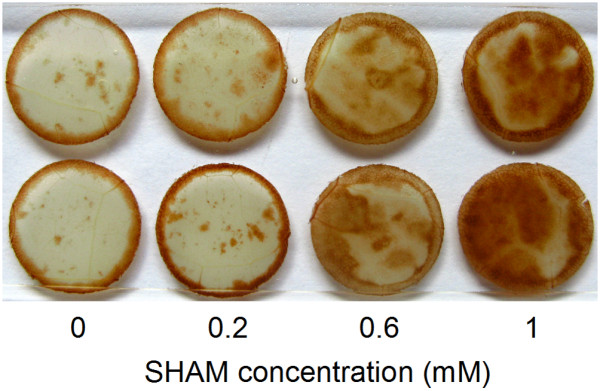
**The accumulation of H_2_O_2 _in *Rumex *K-1 leaves treated with 0 (control), 0.2, 0.6 or 1 mM SHAM under intense light**. Histochemical detection of H_2_O_2 _production with 1 mg ml^-1 ^3, 3-diaminobenzidine (DAB, pH 3.8) staining. Representative images from five independent experiments were shown in the figure.

To visualise H_2_O_2 _accumulation, we stained the leaves with 3, 3-diaminobenzidine (DAB). The inhibition of the AOX pathway by SHAM significantly enhanced the generation of H_2_O_2 _under intense light, and the accumulation of H_2_O_2 _increased significantly with the increase in the SHAM concentration (Figure 11), which indicates that the SHAM-treated *Rumex *K-1 leaves suffered more from photo-oxidative stress compared with control leaves under intense light.

## Discussion

The excess reducing equivalents generated in the chloroplasts under intense light can be transported to other organelles via the malate-OAA shuttle and oxidised in metabolic pathways [17,19,44]. NADP-MDH is a key enzyme in the malate-OAA shuttle [16,19,44]. It is noteworthy that the initial activity of NADP-MDH noticeably increased in *Rumex *K-1 leaves under intense light (Figure 3 B), indicating that the malate-OAA shuttle was activated to transport the excess reducing equivalents generated in the chloroplasts to other organelles. These equivalents transported from chloroplasts can be oxidised by the respiratory electron transport chain [16,44]. It is suggested that in this situation, the AOX pathway, which is a non-phosphorylating electron transport pathway, plays a role in the dissipation of the chloroplast-derived reducing equivalents [16,27]. The fact that the AOX capacity is significantly increased (Figure 3 A) under intense light supports this suggestion.

SHAM, a well-known inhibitor of the mitochondrial AOX pathway, has been widely used to address the interaction between the chloroplasts and the mitochondria in either mesophyll protoplasts or intact tissues [25,26,31-33,45,46]. The 0 (control), 0.2, 0.6 and 1 mM SHAM not affecting Φ_PSII_, qP and NPQ in the isolated intact chloroplasts in the leaves of *Rumex *K-1 (Figure 2), together with the SHAM treatments not altering the OJIP transients (Figure 4 A, D) in the dark, demonstrates that the concentrations of SHAM used in this study had no direct effects on photosynthetic behaviours, which was also previously documented by Padmasree and Raghavendra [31], Bartoli et al. [26] and Yoshida et al. [25] in their studies with other plant materials. Therefore, all of the effects caused by the SHAM treatments in the study actually resulted from the inhibition of the mitochondrial AOX pathway. Furthermore, the SHAM treatments did not change the initial activity of NADP-MDH in the dark (Figure 3 B), which indicates that the concentration of SHAM had no direct effect on the initial activity of NADP-MDH.

It has been reported that, when reducing equivalents accumulate in the mitochondria, the activity of the malate-OAA shuttle will be reduced by a feedback mechanism [47]. Therefore, it is reasonable to consider that, under intense light, the significant decrease in the initial activity of NADP-MDH by the inhibition of the AOX pathway (Figure 3 B) was due to the accumulation of reducing equivalents in the mitochondria because the consumption of reducing equivalents via the AOX pathway was inhibited. The significant decrease in the initial activity of NADP-MDH would inevitably limit the exportation of reducing equivalents from the chloroplasts to the mitochondria, resulting in the accumulation of reducing equivalents in the chloroplast stroma. The SHAM treatments pronouncedly altered the OJIP transients under intense light (Figure 4 B), suggesting that the inhibition of the AOX pathway significantly influenced the performance of the photosynthetic machinery. The fact that P700 was more reduced in the SHAM-treated leaves under intense light compared with control leaves (Figure 5) indicates that the inhibition of the AOX pathway caused an over-reduction of the PSI acceptor side under intense light due to the accumulation of excess reducing equivalents in the chloroplasts. Additionally, according to the JIP-test, the relative variable fluorescence at the J-step increased significantly with the increase in the SHAM concentration (Figure 4 C), indicating that the acceptor side of PSII was more reduced [34,48] under intense light. As a consequence, the photochemical quenching coefficient (qP) decreased, and the photosynthetic linear electron transport flow was restricted, the latter of which was indicated by the significant decreases in the ETR and the photosynthetic O_2 _evolution rate (Figure 6).

It should be emphasised that the electron fluxes between the photosynthetic ETR (Figure 6 A) and the mitochondrial AOX pathway (Figure 3 A) are noticeably different. The generation of reducing equivalents by photosynthesis is over 20 times higher than the consumption by the AOX pathway. Therefore it is difficult to understand how such a low electron flux via the AOX pathway can become a significant sink for photosynthetic electrons. In this experiment, the photosynthetic ETR was determined in the leaf discs exposed to air, but the respiratory rate was determined in the leaf discs submerged in solution. Thus, the significant difference between the photosynthetic electron transport flow and the mitochondrial AOX pathway electron flux could be partially due to the different determination conditions. The results of the experiment determining the photosynthetic O_2 _evolution rate (Figure 6 C) further support the above suggestion. Therefore, the electron flux of the AOX pathway may be underestimated in the experimental conditions. In addition, Dinakar et al. [46] suggested that the restriction of electron transport through the mitochondrial AOX pathway in the cellular environment is simultaneously coordinated with a decline in photosynthesis by the production of biochemical signals, even though the flux of the AOX pathway is very low. Therefore, the inhibition of the AOX pathway down-regulated the photosynthetic electron flow not only by the loss of the sink of excess reducing equivalents but also by the production of certain biochemical signals.

The induction of qE (the fast component of NPQ) requires a thylakoid ΔpH generated by the photosynthetic linear or/and cyclic electron transport to activate the de-epoxidation of violaxanthin to zeaxanthin [3,8,9,12,13]. In the present work, the observation that ms-DLE and ΔPRI decreased gradually with the increase in the SHAM concentration under intense light (Figure 7) suggests that the restriction of the photosynthetic linear electron flow due to the inhibition of the AOX pathway limited the generation of the thylakoid ΔpH and the de-epoxidation of violaxanthin to zeaxanthin. Therefore, the induction of the major and fast component of NPQ, qE, was suppressed in the SHAM-treated leaves (Figure 8 B) because of the restriction of the de-epoxidation of the xanthophyll cycle. In this instance, the slow component of NPQ, qI, was induced to dissipate excess light energy under intense light (Figure 8 C). The restriction in NPQ induction due to the inhibition of the AOX pathway (Figure 8 A) was mainly attributable to the suppression of qE. The restriction in NPQ induction under intense light was also observed by Zhang et al. [49] in *Arabidopsis aox1a *mutant, which further supports the above observation. In this way, a close link between the induction of NPQ and the AOX pathway is established.

However, the detailed mechanisms of how the AOX pathway generates an additional ΔpH under excess light are still unclear, as under excessive light, the ΔpH across the thylakoid membrane is not only because of an accumulation of reduced NADPH but also because of the limiting concentrations of ADP. Further studies are needed to address this question.

To further confirm the role of the AOX pathway, we examined the effects of the inhibition of the AOX pathway on NPQ induction at a higher NaHCO_3 _concentration (20 mM NaHCO_3_). The fact that the effects of the SHAM treatments on the shape of the chlorophyll *a *fluorescence transients (Figure 4 E, F) were less pronounced at 20 mM NaHCO_3 _than at 1 mM NaHCO_3 _indicates that higher concentration of NaHCO_3 _might maintain the photosynthetic electron transport chain in a more oxidised state to allow the photosynthetic linear electron flow to move faster because a higher concentration of the intercellular CO_2 _acted as an efficient electron acceptor. This idea was also demonstrated by Padmasree and Raghavendra [31] and by our pre-experiment work showing that the photosynthetic O_2 _evolution rate in *Rumex *K-1 leaf discs was significantly higher at 20 mM NaHCO_3 _than at 1 mM NaHCO_3 _(data not shown). Hence, the effect of the inhibition of the AOX pathway on the photosynthetic behaviours at 20 mM NaHCO_3 _was smaller than that at 1 mM NaHCO_3_. The observation that the effects of the SHAM treatments on the induction of NPQ (Figure 8 D), qE (Figure 8 E) and qI (Figure 8 F) at 20 mM NaHCO_3 _were less than the effects at 1 mM NaHCO_3 _under intense light further supports the viewpoint that the AOX pathway plays an important role in the induction of NPQ. All of these results suggest that the AOX pathway can decrease the extent of the reduction of the PSI acceptor side and the extent of the excitation of the PSII reaction centres through the dissipation of excess reducing equivalents in chloroplasts and the induction of NPQ.

Furthermore, during the induction phase of photosynthesis, the increases in the ETR (Figure 9 A) and qP (Figure 9 B) were restricted by various concentrations of SHAM after two minutes of illumination, which suggests that the AOX pathway is also essential for the maintenance of the photosynthetic linear electron transport flow during the induction phase of photosynthesis. Consequently, the inhibition of the AOX pathway suppressed the development of NPQ after two minutes of illumination (Figure 9 C). Meanwhile, after the leaves were transferred from intense light to darkness, the differences in the level of NPQ among the leaves treated with various concentrations of SHAM were abolished within one minute (Figure 9 C). Because qE, but not qI is quickly quenched after the leaves are transferred to darkness [3], the restriction in NPQ development due to the inhibition of the AOX pathway is attributable to the suppression of qE formation. Therefore, during the induction phase of photosynthesis, the AOX pathway should also be required to produce a thylakoid ΔpH and drive the de-epoxidation of violaxanthin to induce NPQ. This possibility is supported by the report that NADP-MDH, the key enzyme of the malate-OAA shuttle, is almost fully activated within two minutes of illumination in dark-adapted mesophyll protoplasts of pea [47].

A substantial accumulation of H_2_O_2 _was observed in the SHAM-treated leaves under intense light (Figure 11), which suggests that the AOX pathway may function as an antioxidant mechanism to suppress the generation of ROS. It has been known that when PSI is over-reduced, more ROS will be produced at the acceptor side of PSI. Based on the above results, we assume that the AOX pathway suppresses the generation of ROS maybe by alleviating the over-reduction of the PSI acceptor side and accelerating the induction of NPQ.

It has been suggested that ROS can induce the inactivation of the repair of the photodamaged PSII by suppressing the de novo synthesis of the D1 protein [50-52], which therefore limits the photosynthetic ETR. Additionally, our recent study [53] showed that the AOX pathway protects plants against photoinhibition by alleviating the inhibition of the repair of the photodamaged PSII through preventing the formation of ROS in *Rumex *K-1 leaves. Therefore, the enhancement of photoinhibition by the SHAM treatment is most likely partially caused by the inhibition of the repair of the photoinhibited PSII by H_2_O_2_. It could be argued that the suppression of the NPQ induction is due to the production of ROS. Compared with the control leaves, the development of NPQ was suppressed after two minutes of illumination (Figure 9 C). However, the extent of the photoinhibition was accelerated after 5 minutes of illumination (Figure 10) in the SHAM-treated leaves, suggesting that the suppression of the NPQ induction is at least partially due to the inhibition of the AOX pathway by SHAM under photoinhibition conditions. Dinakar et al. [46] indicated that any perturbation in the capacities of the AOX or COX pathway of mitochondrial oxidative electron transport in light leads to disturbances in the maintenance of photosynthesis through modulations of ROS, antioxidant enzymes and antioxidant molecules. These authors suggested that ROS and antioxidant metabolites could act as biochemical signals during the beneficial interactions of the mitochondrial metabolism with photosynthesis. Therefore, we do not disregard the side effects of ROS production on photosynthetic electron transport and on the induction of NPQ after the occurrence of serious photoinhibition. However, further investigation is required to clarify how the restriction in electron transport through the AOX pathway or the COX pathway of mitochondria in the cellular environment is simultaneously coordinated with the decline in photosynthesis through changes in ROS and different components of the antioxidant system.

## Conclusions

**Figure 12:**
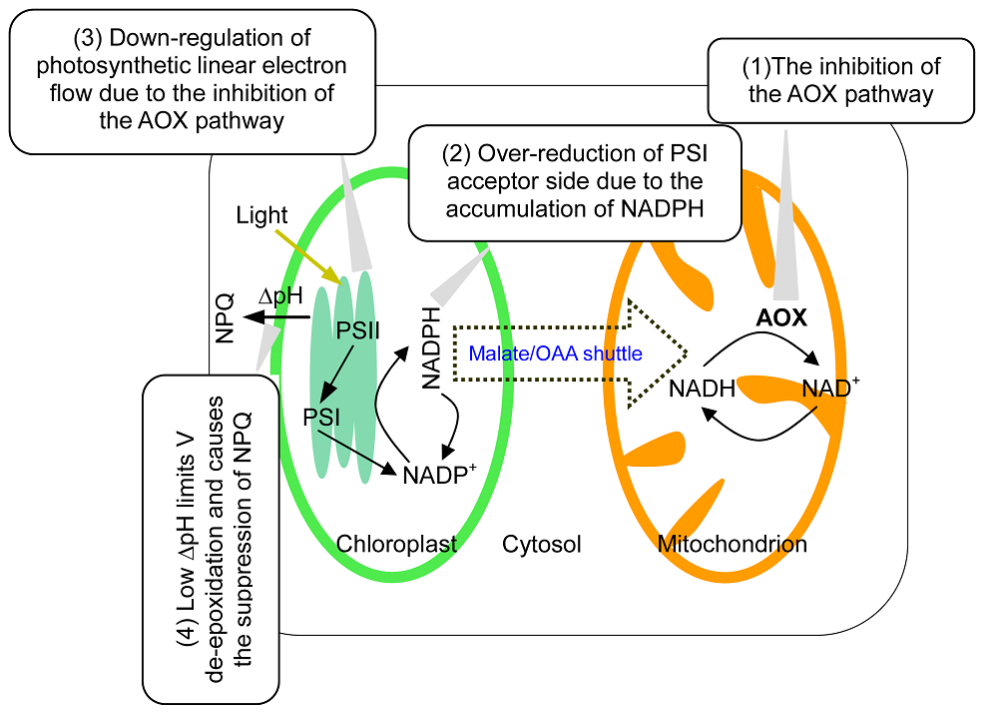
**Schemes of the possible phenomena in the cell when the AOX pathway is inhibited in the high light**. AOX, alternative oxidase; NPQ, non-photochemical quenching; OAA, oxaloacetate; PSII/I, photosystem II/I; V, violaxanthin.

The inhibition of the AOX pathway resulted in the rapid accumulation of NADPH in the chloroplasts, leading to the over-reduction of the PSI acceptor side. Furthermore, the restriction of the photosynthetic linear electron flow due to the inhibition of the AOX pathway inevitably limited the generation of the thylakoid ΔpH, which thus limited the de-epoxidation of violaxanthin and further suppressed the induction of NPQ (Figure 12). Therefore, the mitochondrial AOX pathway protects the photosynthetic apparatus against photodamage by alleviating the over-reduction of the PSI acceptor side and accelerating the induction of NPQ.

## Methods

### Plant materials and treatments

*Rumex *K-1 plants (*Rumex patientia *× *R. tianschaious*) were grown from seed under a 14-h photoperiod at 22/18°C (day/night) in pots containing soil. The plants were thinned to one plant per pot two weeks after sowing. Sufficient nutrients and water were supplied throughout cultivation to avoid any potential nutrient or drought stresses. The photon flux density (PFD) during growth was approximately 600 μmol m^-2 ^s^-1^. The fully expanded leaves of 4-week-old plants were used in the experiments.

Intact chloroplasts were isolated according to the protocol of Bartoli et al. [26]. Leaves were ground in a buffer containing 50 mM HEPES-KOH (pH 7.5), 330 mM sorbitol, 2 mM Na_2_EDTA, 1 mM MgCl_2_, 5 mM ascorbic acid, and 0.05% (w/v) BSA using a hand-held homogeniser. The homogenate was filtered through a 20 μm pore size nylon mesh and centrifuged at 3000 g for 5 minutes. The pellet was suspended in wash medium (50 mM HEPES-KOH pH 7.5 and 330 mM sorbitol), loaded in a solution consisting of 35% (v/v) Percoll in wash medium, and centrifuged at 2500 g for 5 minutes. The pellet containing the intact chloroplasts was used to measure the modulated chlorophyll fluorescence parameters in the presence of 0 (control), 0.2, 0.6 or 1 mM SHAM. The isolated chloroplasts were 75% intact according to the ferricyanide-dependent O_2 _evolution [54] with a Chlorolab-2 oxygen electrode (Hansatech Instruments, Norfolk, UK).

Discs (0.5 cm^2^) were punched from fully expanded leaves and vacuum-infiltrated with 0 (control), 0.2, 0.6 or 1 mM SHAM solution combined with 1 mM NaHCO_3 _or 20 mM NaHCO_3 _for 2 h in the dark at room temperature. Then, the discs, adaxial side up, were submerged in the corresponding concentration of SHAM combined with NaHCO_3 _solution. Next, the discs were exposed to intense light measuring 800 μmol m^-2 ^s^-1 ^for 30 min at room temperature, and air was supplied for 10 s every 10 min from the bottom of containers during intense light treatment.

In addition, discs (1.3 cm^2^) were punched from fully expanded leaves and exposed to light measuring 100, 300, 500, 700 and 1000 μmol m^-2 ^s^-1 ^for 1 h at room temperature. The discs were used to measure the de-epoxidation of the xanthophyll cycle (ΔPRI) and the components of the xanthophyll cycle. Other discs (1.3 cm^2^) were punched from fully expanded leaves and infiltrated with 0 (control), 0.2, 0.6 or 1 mM SHAM solution combined with 1 mM NaHCO_3 _for 2 h in the dark at room temperature. Then, the discs, adaxial side up, were exposed to intense light measuring 800 μmol m^-2 ^s^-1 ^for 0, 2, 5, 10, 30 min. The discs were used to measure the maximum quantum yield of photosystem II (F_v_/F_m_).

### Capacity of AOX pathway measurement

The respiratory rate in the leaf discs treated with intense light was measured using an Oxytherm oxygen electrode (Hansatech Instruments, Norfolk, UK) at 25°C according to Yoshida et al. [25] and Zhang et al. [49]. The leaf discs were incubated in the dark for 10 minutes before the measurement of respiration. The inhibitors of the COX (1 mM KCN) and AOX pathways (20 mM SHAM) were used to inhibit the respective pathways. The AOX pathway capacity was defined as the O_2 _uptake rate in the presence of KCN that was sensitive to salicylhydroxamic acid.

### Enzyme assay

NADP-malate dehydrogenase (NADP-MDH) was extracted from the leaves according to Dutilleul et al. [55]. Leaf discs (7.5 cm^2^) were ground in liquid N_2 _and extracted into 50 mM HEPES-KOH (pH 7.5) buffer containing 10 mM MgCl_2_, 1 mM Na_2_EDTA, 5 mM dithiothreitol (DTT), a protease inhibitor tablet, 5% (w/v) insoluble polyvinylpyrrolidone and 0.05% (v/v) Triton X-100. After centrifugation for 5 minutes at 14,000 g, the enzymatic activity in the supernatant was measured with an UV-2550 spectrophotometer (Shimadzu, Japan). The initial activity of NADP-malate dehydrogenase was measured according to Dutilleul et al. [55]. The assay was performed in 40 mM Tricine-KOH (pH 8.3), 150 mM KCl, 1 mM Na_2_EDTA, 5 mM DTT, 0.2 mM NADPH and 2 mM OAA, plus the sample.

### Chlorophyll *a *fluorescence transient measurement

The chlorophyll *a *fluorescence (OJIP) transient was measured using a Handy Plant Efficiency Analyser (Hansatech Instruments, Norfolk, UK). The transient was induced by a red light measuring about 3000 μmol m^-2 ^s^-1 ^provided by an array of three light-emitting diodes (peak 650 nm). All the measurements were performed with 15 minute dark-adapted leaf discs at room temperature.

The OJIP transient was analysed according to the JIP-test [34,39-41,48,56,57] by using the following original data: (a) the fluorescence intensity at 50 μs considered to be F_o _when all of the PSII RCs are open and (b) the maximal fluorescence intensity, F_m_, assuming that the excitation intensity is high enough to close all of the RCs of PSII. The relative variable fluorescence at any time, V_t _= (F_t_-F_o_)/(F_m_-F_o_) and the difference in the kinetics of OJIP transients between the control leaves and the SHAM-treated leaves, ΔV_t _= Vt_(various concentrations of SHAM-treated leaves)_-V_t(control leaves)_.

### P700 redox state measurement

The redox state of P700 was measured according to Schansker et al. [58], Kim et al. [59] and Zhang et al. [53] using a PEA Senior fluorometer (Hansatech Instruments, Norfolk, UK). Red light, measuring 800 μmol m^-2 ^s^-1^, was produced by an array of four 650 nm light-emitting diodes (LED, peak 650 nm), and the modulated (33.3 kHz) far-red measuring light has a wavelength of 820 nm was provided by an OD820 LED (Opto Diode Crop., USA). Upon irradiation with a red pulse (800 μmol m^-2 ^s^-1^), the transmission at 820 nm in leaves increases gradually, which is mainly caused by the reduction of P700^+^. And the signal was recorded when the reduction of P700^+ ^attained steady-state levels.

### Photosynthetic O_2 _evolution rate measurement

A Chlorolab-2 liquid-phase oxygen electrode system (Hansatech Instruments, Norfolk, UK) was used to measure the photosynthetic O_2 _evolution rates of *Rumex *K-1 leaf discs in 1 mM NaHCO_3 _solution under saturation light (800 μmol m^-2 ^s^-1^) at room temperature.

### The components of the xanthophyll cycle measurements

The components of the xanthophyll cycle were measured according to Thayer and Björkman [60]. *Rumex *K-1 leaves treated with different light intensities were frozen in liquid nitrogen and extracted with 100% acetone. Pigment separation was performed in an HPLC system (Waters, USA). The actual de-epoxidation status of the xanthophyll cycle pigment pool was calculated: (A + Z)/(V + A + Z). Before freezing the leaf samples, the de-epoxidation of the xanthophyll cycle, expressed as ΔPRI, was measured with a Unispec portable spectrometer combined with bifurcated fibre optics and a leaf clip (PP Systems, USA). After the chemical analysis of the components of the xanthophyll cycle, the correlation between the ΔPRI and (A + Z)/(V + A + Z) in the leaves of *Rumex *K-1 was established.

### Ms-DLE measurement

The measurements of the ms-DLE were conducted using an M-PEA fluorometer (Hansatech Instruments, Norfolk, UK) according to Wang et al. [42] with modifications. The sample was irradiated with red light produced by an array of four 650 nm light-emitting diodes (LED, peak 650 nm), and the measuring process was divided into consecutive cycles of 100 ms of excitation by light followed by 23 ms of darkness. The DLE between 2.8 and 4 ms after every flash was measured with an M-PEA fluorometer, and the signal was recorded continuously by a computer.

### Modulated chlorophyll fluorescence parameters measurements

The modulated chlorophyll fluorescence parameters of leaf discs were measured with a FMS-2 pulse-modulated fluorometer (Hansatech Instruments, Norfolk, UK) as described in Jiang et al. [61]. And the modulated chlorophyll fluorescence parameters of the intact *Rumex *K-1 chloroplasts were measured with the FMS-2 pulse-modulated fluorometer (Hansatech Instruments, Norfolk, UK) integrated with a Chlorolab-2 oxygen electrode (Hansatech Instruments, Norfolk, UK). The actinic light measuring 800 ml m^-2 ^s^-1 ^was offered by the light source of Chlorolab-2. The measurements of F_o _and F_m _were done with leaf discs and chloroplasts which were treated with various concentrations of SHAM in the dark. The steady-state fluorescence level (F_S_), the light-adapted minimum (F_o_^'^) and maximum (F_m_^'^) fluorescence of leaf discs were also measured after illumination.

The following parameters were then calculated: (1) the maximum quantum yield of photosystem II, F_v_/F_m _= (F _m_-F_o_)/F_m_; (2) the photochemical quenching coefficient, qP = (F_m_^'^-F_S_)/(F_m_^'^-F_o_^'^) (3) The PSII electron transport rate, ETR = 0.84·0.5·Φ_PSII_·PFD, where Φ_PSII _(photosystem II actual photochemical efficiency) = 1-F_S_/F_m_^'^; (4) Non-photochemical quenching, NPQ = (F_m_-F_m_^'^)F_m_^'^.

Components of NPQ, including a fast component (qE) and a slow component (qI), were determined following the protocol of Johnson et al. [62].The induction kinetics of modulated chlorophyll fluorescence parameters were measured by illuminating the dark-adapted leaf discs with high light (800 μmol m^-2 ^s^-1^).

### De-epoxidation of xanthophyll cycle measurement

The de-epoxidation of xanthophyll cycle expressed by ΔPRI of leaves during photosynthetic induction was measured with a Unispec portable spectrometer combined with bifurcated fibre optics and a leaf clip (PP Systems, USA). The leaf clip held the fiber at a 60° angle to the leaf discs. Leaf irradiation measuring about 800 μmol m^-2 ^s^-1 ^was provided through one side of the bifurcated fibre from a halogen lamp in the spectrometer. Leaf photochemical reflectance index were then calculated from the reflectance data as follows [63,64]: PRI = (R_531_-R_570_)/(R_531 _+ R_570_), where R refers to reflectance, and the subscript refers to the wavebands in nanometers. And the de-epoxidation of xanthophyll cycle ΔPRI = PRI_(high light)_-PRI_dark_.

### Histochemical detection of H_2_O_2_

Leaf discs (1.3 cm^2^) were vacuum-infiltrated with 1 mg ml^-1 ^3,3-diamino-benzidine (DAB, pH 3.8) and incubated under intense light (800 μmol m^-2 ^s^-1^) conditions for 2 h after treatment with 0 (control), 0.2, 0.6 and 1 mM SHAM respectively in the dark for 2 h. After photoinhibition treatment, leaf discs were decolorized by immersion in boiling ethanol (96%) for 10 minutes. This treatment decolorized the leaves except for the deep brown polymerization product produced by the reaction of DAB with H_2_O_2_. After cooling, the leaf discs were extracted at room temperature with fresh ethanol and photographed [65].

### Statistical analysis

LSD (least significant difference) was used to analyse differences between the SHAM treatments by using SPSS 16.

## Abbreviations

A: Antheraxanthin; AOX: Alternative oxidase; CEF: Cyclic electron flow; COX: Cytochrome oxidase; DAB: 3: 3-diaminobenzidine; ETR: PSII electron transport rate; Fd: Ferredoxin; F_v_/F_m_: Maximal quantum yield of PSII; H_2_O_2_: Hydrogen peroxide; LED: Light-emitting diodes; Mal: Malate; ms-DLE: Ms-delayed light emission; NADP-MDH: NADP-Malate dehydrogenase; NPQ: Non-photochemical quenching; OAA: Oxaloacetate; OJIP: Chlorophyll *a *fluorescence transient; PFD: Photon flux density; PQ: Plastoquinone; PSI/II: Photosystem I/II; qE: Fast component of NPQ; qI: Slow component of NPQ; qP: Photochemical quenching coefficients; ROS: Reactive oxygen species; SHAM: Salicylhydroxamic acid; UQ: Ubiquinone; V: Violaxanthin; WWC: Water-water cycle; Z: Zeaxanthin; ΔPRI: De-epoxidation of the xanthophyll cycle; Φ_PSII_: Actual PSII photochemical efficiencies.

## Authors' contributions

LTZ performed most of the experiments and wrote the manuscript. HYG, JGL and QWM designed and directed the study and revised the manuscript. ZSZ and XLM helped in measuring OJIP transients. CY helped in assaying NADP-MDH initial activity. All authors have read and approved the final manuscript.
